# Complex chemical composition of colored surface films formed from reactions of propanal in sulfuric acid at upper troposphere/lower stratosphere aerosol acidities

**DOI:** 10.5194/acp-15-4225-2015

**Published:** 2015-04-24

**Authors:** A. L. Van Wyngarden, S. Pérez-Montaño, J. V. H. Bui, E. S. W. Li, T. E. Nelson, K. T. Ha, L. Leong, L. T. Iraci

**Affiliations:** 1 Department of Chemistry, San José State University, San José, CA 95192, USA; 2 Atmospheric Science Branch, NASA Ames Research Center, Moffett Field, CA 94035, USA

## Abstract

Particles in the upper troposphere and lower stratosphere (UT/LS) consist mostly of concentrated sulfuric acid (40–80 wt %) in water. However, airborne measurements have shown that these particles also contain a significant fraction of organic compounds of unknown chemical composition. Acid-catalyzed reactions of carbonyl species are believed to be responsible for significant transfer of gas phase organic species into tropospheric aerosols and are potentially more important at the high acidities characteristic of UT/LS particles. In this study, experiments combining sulfuric acid (H_2_SO_4_) with propanal and with mixtures of propanal with glyoxal and/or methylglyoxal at acidities typical of UT/LS aerosols produced highly colored surface films (and solutions) that may have implications for aerosol properties. In order to identify the chemical processes responsible for the formation of the surface films, attenuated total reflectance–Fourier transform infrared (ATR-FTIR) and ^1^H nuclear magnetic resonance (NMR) spectroscopies were used to analyze the chemical composition of the films. Films formed from propanal were a complex mixture of aldol condensation products, acetals and propanal itself. The major aldol condensation products were the dimer (2-methyl-2-pentenal) and 1,3,5-trimethylbenzene that was formed by cyclization of the linear aldol condensation trimer. Additionally, the strong visible absorption of the films indicates that higher-order aldol condensation products must also be present as minor species. The major acetal species were 2,4,6-triethyl-1,3,5-trioxane and longer-chain linear polyacetals which are likely to separate from the aqueous phase. Films formed on mixtures of propanal with glyoxal and/or methylglyoxal also showed evidence of products of cross-reactions. Since cross-reactions would be more likely than self-reactions under atmospheric conditions, similar reactions of aldehydes like propanal with common aerosol organic species like glyoxal and methylglyoxal have the potential to produce significant organic aerosol mass and therefore could potentially impact chemical, optical and/or cloud-forming properties of aerosols, especially if the products partition to the aerosol surface.

## 1 Introduction

Aerosols in the upper troposphere and lower stratosphere (UT/LS) are composed primarily of sulfuric acid (40–80 wt %) (Clegg et al., 1998; Finlayson-Pitts and Pitts, 2000; Tabazadeh et al., 1997) and water, but they also contain significant fractions of organic compounds (Froyd et al., 2009; Murphy et al., 2007, 2014, 1998). In the case of UT aerosols, the amount of organic material can even exceed the amount of sulfate present (Murphy et al., 1998). The potential impacts of this organic material on chemical, optical and cloud-forming properties of UT/LS aerosols are highly uncertain since relatively little is known about the chemical composition of the organic fraction because available sampling techniques and frequencies are limited by the high-altitude airborne missions required.

In contrast to UT/LS aerosols, tropospheric aerosols are better sampled so it is well established that they contain major fractions of organics (up to 90 %) (e.g., Calvo et al., 2013; Hallquist et al., 2009; Jacobson et al., 2000; Jimenez et al., 2009; Kanakidou et al., 2005; Murphy et al., 2006; Zhang et al., 2007), and there have been many studies aimed at chemical characterization of tropospheric organic aerosol (OA) particles and at determining the physical/chemical pathways for the formation of OA. In particular, reactions of carbonyl-containing organic species including aldol condensation, hemiacetal/acetal formation, organosulfate formation and various polymerization reactions have all been identified as potential sources of low-volatility organic products in tropospheric organic aerosols (Barsanti and Pankow, 2004; Ervens and Volkamer, 2010; Gao et al., 2004; Garland et al., 2006; Holmes and Petrucci, 2007; Jang et al., 2002, 2004; Kalberer et al., 2004; Liggio and Li, 2006, 2008; Liggio et al., 2007; Lim et al., 2010; Michelsen et al., 2004; Nozière and Esteve, 2007; Nozière and Riemer, 2003; Sareen et al., 2010; Shapiro et al., 2009; Surratt et al., 2007, 2006; Tan et al., 2010; Tolocka et al., 2004; Zhao et al., 2005; Ziemann and Atkinson, 2012). Since these reactions are all either acid-catalyzed or require sulfate, they are likely to be even more favorable at the high sulfuric acid concentrations typical of UT/LS aerosols.

Preliminary experiments for the current work, in which various carbonyl species (propanal, glyoxal and/or methylglyoxal) were combined with highly concentrated sulfuric acid to simulate UT/LS aerosol acidities, produced highly colored solutions; solutions containing propanal also produced reaction products that partitioned to the liquid surface as macroscopic semi-solid surface films that were also highly colored. The possibility that similar organic products could partition to thin layers or films on the surface of UT/LS aerosols is of particular interest because organic compounds that coat aerosol particles would have the most dramatic effects on aerosol chemical, optical and/or cloud-forming properties (see Donaldson and Vaida, 2006, and McNeill et al., 2013, for reviews of aerosol surface coatings and their impacts on aerosol properties). For example, organic coatings on aqueous droplets and sulfuric acid aerosols have been observed to impede water uptake and/or evaporation in laboratory experiments (e.g., Davies et al., 2013; Otani and Wang, 1984; Rubel and Gentry, 1984; Seaver et al., 1992; Xiong et al., 1998), so organic coatings on UT/LS aerosols and/or droplets could potentially inhibit water condensation and therefore cloud formation and/or growth. Organic coatings may also impact heterogeneous reactions at aerosol surfaces; for example, reactive uptake of N_2_O_5_ has been shown to be impeded by various organic coatings which could reduce the rate of hydrolysis of N_2_O_5_ to HNO_3_ on sulfuric acid aerosols, affecting NO*_x_* and OH budgets (Anttila et al., 2006; Badger et al., 2006; Cosman and Bertram, 2008; Cosman et al., 2008; Escorcia et al., 2010; Evans and Jacob, 2005; Folkers et al., 2003; Gaston et al., 2014; Knopf et al., 2007; McNeill et al., 2006; Park et al., 2007; Riemer et al., 2009; Thornton and Abbatt, 2005). Similarly, organic coatings on sulfate aerosols would alter optical properties, especially if the organics are highly absorbing in the UV–visible. In order to assess whether species that form surface films on propanal/H_2_SO_4_ mixtures in the laboratory could be important in UT/LS aerosols, the reactions responsible for film formation must be identified, which is, therefore, the focus of the present work.

Recent work with various other aldehydes (Li et al., 2011; Sareen et al., 2010; Schwier et al., 2010) demonstrated that products of reactions of formaldehyde, acetaldehyde, glyoxal, methylglyoxal and their mixtures are surface-active even in water and ammonium sulfate/water solutions characteristic of less acidic lower tropospheric aerosols. Their chemical characterization of the reaction products identified hemiacetal oligomers and aldol condensation products, but the surface-active species were not specifically identified.

**Figure 1:**
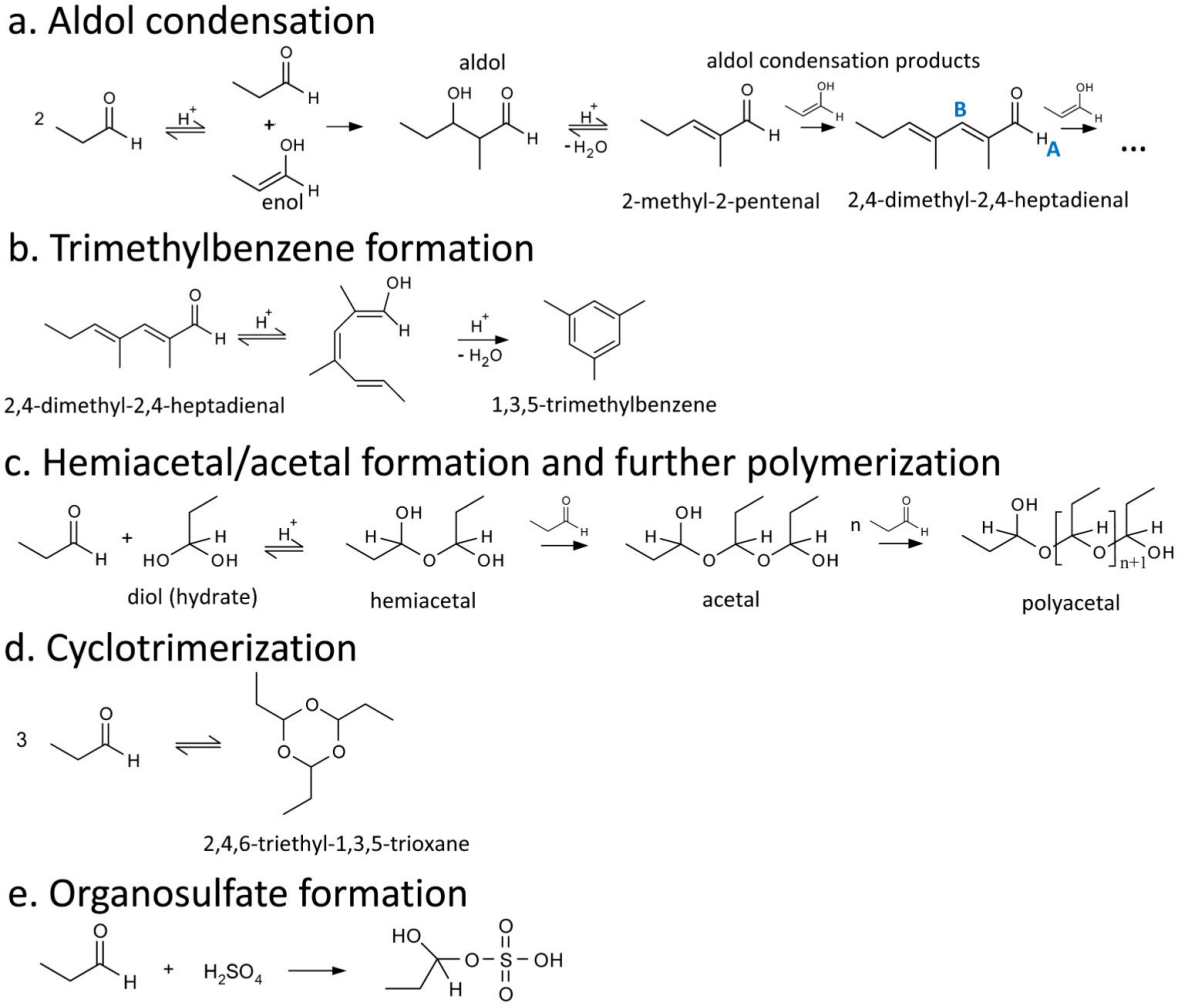
Potential reactions of propanal in the presence of sulfuric acid. Selected hydrogen positions are labeled A and B in blue to facilitate discussion in the text.

In order to identify the chemical species present in films formed by propanal and sulfuric acid, we consider the products of the following potential reactions (identified by a letter in Fig. 1): (a) aldol condensation, (b) trimethylbenzene formation via cyclization of the linear trimer produced by aldol condensation, (c) hemiacetal, acetal and/or polyacetal formation, (d) trioxane formation via cyclotrimerization and (e) organosulfate formation. Each of these processes result in higher molecular weight products, which could result in partitioning to the solid phase as a surface film.

Aldol condensation products are expected since it can be seen from Fig. 1 that they are the only potential products containing sufficient conjugation to absorb visible light, but they are not necessarily the major component of the films since only tiny amounts of such chromophores are necessary for color (McLaren, 1983). Products of propanal aldol condensation reactions have been observed in aqueous media containing various catalysts, including anion exchange resin (Pyo et al., 2011), ammonium and carbonate salts (Nozière et al., 2010), mixed metal oxides (Tichit et al., 2002) and zeolites (Hoang et al., 2010). In the case of zeolite catalysts, 1,3,5-trimethylbenzene was also observed and proposed to form from the linear trimer produced by aldol condensation reactions (Fig. 1b). Aldol condensation reactions of propanal have also been studied in concentrated sulfuric acid (60–96 wt %) solutions by Nozière and Esteve (2007) and Casale et al. (2007). Nozière and Esteve (2007) reported the UV–visible spectra of aldol condensation products of six carbonyl compounds including propanal and concluded that their absorption index could become significant over the approximately 2-year residence time of stratospheric aerosols. Casale et al. (2007) measured bulk reaction rates for a series of aliphatic aldehydes (C_2_–C_8_), showing that butanal and propanal had the highest reaction rates but concluding that the rates were not fast enough to be responsible for transfer of significant organic mass into tropospheric aerosols. Both studies focused on aldol condensation reactions due to their potential to form light absorbing compounds and therefore used UV–visible spectroscopy for product detection, which is not sensitive to products of the other potential reactions (Fig. 1c–e) considered here.

Propanal may also undergo acid-catalyzed reactions with its hydrated form (diol) to form hemiacetals, acetals and/or linear polyacetals as shown in Fig. 1c. In addition to these linear species, propanal may also undergo acid-catalyzed cyclotrimerization to form a cyclic polyacetal (a trioxane) (Fig. 1d). These reactions have not been reported specifically for propanal in sulfuric acid, but Garland et al. (2006) have shown that sulfuric acid aerosols exposed to hexanal vapor contained hemiacetals, while Li et al. (2008) identified a trioxane in bulk reactions of octanal with sulfuric acid (but not in sulfuric acid aerosols exposed to octanal vapor). In both studies, aldol condensation products were also observed. Furthermore, propanal has been shown to form a trioxane in aqueous solution (Corrochano et al., 2010) and to form a mixture of aldol condensation products, hemiacetals and acetals in the presence of an anion-exchange resin catalyst (Pyo et al., 2011).

Lastly, alcohols may react with sulfuric acid to form sulfate esters (Deno and Newman, 1950; Iraci et al., 2002; Michelsen et al., 2006; Minerath et al., 2008; Van Loon and Allen, 2004, 2008; Vinnik et al., 1986), so alcohol species including the diol formed by hydration of propanal and/or (hemi-)acetals (Surratt et al., 2008) formed from propanal (Fig. 1c) could react directly with sulfuric acid to form organosulfates similar to those formed by reaction of glyoxal on sulfuric acid aerosols (Liggio et al., 2005). An example is shown for reaction of the propanal hydrate in Fig. 1e.

In the present study we first employ a combination of attenuated total reflectance–Fourier transform infrared (ATRFTIR) and ^1^H nuclear magnetic resonance (^1^H NMR) spec-troscopies to identify the major species in the films formed by propanal on sulfuric acid solutions. In order to approach more atmospherically realistic mixtures of organics and to address the possibility of cross-reactions between different carbonyl species, we also examined films formed on mixtures of propanal with glyoxal and/or methylglyoxal. Finally, we also used UV–visible spectroscopy of the liquid solutions to gain chemical insight into the identity of the chromophores and to illustrate their potential importance for UT/LS aerosol optical properties.

## 2 Experimental methods

Surface films were first detected on solutions of propanal and its mixtures with glyoxal and/or methylglyoxal in sulfuric acid, which were allowed to react for several weeks (see Fig. S1 in the Supplement for photos of typical surface films). Subsequently, controlled survey studies were performed to examine the conditions required for formation of surface films. In these experiments, samples of propanal, glyoxal and/or methylglyoxal in all possible combinations of 1, 2 or all 3 species (0.030 M in each organic present) were prepared in stock solutions of 19, 37, 48 and 76 wt % sulfuric acid (H_2_SO_4_). Since initial experiments indicated that solutions of glyoxal and/or methylglyoxal did not form films without the presence of propanal (ultimately confirmed by these survey experiments), concentrations of mixed organics were chosen to keep the propanal concentration constant, so that any differences in film formation rates in the mixtures compared to propanal alone could not simply be due to a different concentration of propanal and therefore would indicate that glyoxal and/or methylglyoxal could impact the ability of propanal to form films. This results in samples that have a total organic concentration that increases with the number of organics present up to 0.09 M for solutions that contain all three organics. Although UT/LS aerosol concentrations of these organic compounds are unknown, 0.03 M is likely much larger than UT/LS concentrations of any one carbonyl species but is more reasonable if considered as representative of the total aldehyde or carbonyl concentration. Sulfuric acid stock solutions were prepared by dilution of concentrated sulfuric acid (96–98 wt %, Sigma-Aldrich, ACS grade) with Milli-Q water, and concentrations were confirmed by titration with standardized sodium hydroxide (0.5 N, Sigma-Aldrich). The following Sigma-Aldrich organics were used: 97 wt % reagent grade propanal, 40 wt % glyoxal and 40 wt % methylglyoxal in water. 4.0 mL aliquots of each mixture were transferred to multiple 8 mL glass vials and stored under each of the following temperature and lighting conditions: room temperature (21–24°C)/constant fluorescent light, room temperature/dark, 0°C/dark, −19°C/dark. Samples were visually monitored daily for color changes and formation of surface films in order to survey which mixtures formed films and to assess the impact of acidity, organic mixture, temperature and fluorescent light on film formation rates.

Chemical analysis of the films required production of films in sufficient quantity to allow physical removal of a portion without disturbing the underlying sulfuric acid solutions and thereby avoiding spectroscopic interferences from water and sulfuric acid. Therefore, samples used for chemical analysis were prepared as above, except at the higher concentration of 0.30 M in each organic and were stored in volumetric flasks (room temperature/fluorescent light) which caused the film to concentrate on the small liquid surface area in the neck of the flask. Film samples were removed and transferred with a glass rod to the surface of an ATR crystal for analysis by FTIR spectroscopy. ATR-FTIR spectra of the films and standards were collected on a Nicolet 6700 spectrophotometer from 4000 to 700 cm^−1^ at 1 cm^−1^ resolution using a mercury cadmium telluride (MCT) detector and a 10-bounce AMTIR ATR crystal with 45° mirrors from PIKE. ATR-FTIR was chosen for chemical analysis since the semi-solid films could be directly analyzed on a crystal compatible with concentrated sulfuric acid and without any need to alter the chemical environment by dissolving the sample in a solvent. In order to provide more chemical specificity, films were also analyzed by ^1^H NMR spectroscopy using a Varian INOVA 400 MHz spectrometer. NMR samples were prepared by dissolving film samples in deuterated chloroform (CDCl_3_) in quartz NMR tubes (5 mm outer diameter). ATRFTIR and/or NMR spectra were also recorded for the following commercially available standards: 2-methyl-2-pentenal (97 wt % Sigma-Aldrich), 1,3,5-trimethylbenzene and 2,4,6-triethyl-1,3,5-trioxane (AKos GmbH, custom synthesis).

Finally, the UV–visible absorption spectra of solutions (0.030 M in each organic) were obtained using a Varian Cary 50 Bio UV–visible spectrometer with a diode array detector and quartz cuvettes of various path lengths from 0.01 to 10 mm for different regions of the spectrum. Prior to analysis, solutions were filtered through 2.5 μm Teflon filters to remove any suspended solid particulates.

## 3 Results

### 3.1 Formation of organic surface films

Carbonyl-containing organics (propanal, glyoxal and/or methylglyoxal) mixed with sulfuric acid (19–76 wt %) to simulate UT/LS aerosol acidities produced colored solutions, precipitates and surface films. At the highest acidities, all individual organics and organic mixtures examined (0.030 M in each organic) produced visibly colored solutions that darkened with time. Mixtures containing propanal produced the most deeply colored solutions, progressing from yellow to orange to red to brown over timescales ranging from minutes to months. This color darkening progressed faster at higher acidities, consistent with an acid-catalyzed reaction. Many propanal-containing mixtures also eventually produced colored precipitates and/or surface films (mixtures containing only glyoxal and/or methylglyoxal did not produce surface films). These solids or semi-solids were observed either as particles suspended in the liquid (usually collecting near the surface) and/or as semi-rigid macroscopic films on the surface. In principle, the films could potentially be formed either by heterogeneous reactions at the air/liquid interface or by liquid-phase reactions resulting in products that partition to the surface. The latter process, however, is supported by the observation that when solutions were stored in volumetric flasks solid, dark-colored material sometimes collected on the upper slanted walls in the body of the flask before migrating to the surface; presumably the material rose due to its low density relative to the solution but was temporarily impeded from reaching the surface by the flask walls. Furthermore, the quantity of film material observed cannot be easily explained by heterogeneous surface reactions alone.

There was variability in film formation rates for replicates of the survey experiments most likely due to differences in the gentle movement of the samples that was required to detect films during daily visual observations; however, the following general trends emerged (see Figs. S5 and S6 in the Supplement for trends and variability). First, the precise dependence of film-formation rate on acidity was complex, but, in general, the films formed faster at higher acidity, consistent with acid-catalyzed processes. In fact, the most acidic (76 wt % H_2_SO_4_) propanal/glyoxal mixture produced a surface film immediately upon combining the reactants, although the other organic mixtures formed films more slowly at 76 wt % than at 48 wt % H_2_SO_4_. Specifically, films were first observed on propanal-only samples after 4 days in 48 wt % H_2_SO_4_ vs. 5–10 days in 76 wt % H_2_SO_4_, and visible film formation on propanal/methylglyoxal and propanal/glyoxal/methylglyoxal samples required 5–22 days in 48 wt % H_2_SO_4_, while samples in 76 wt % H_2_SO_4_ still did not have visible films after 180 days. Second, film-formation rates also varied as a function of organic mixture. In general, most mixtures containing glyoxal formed films more rapidly than those without, while mixtures containing methylglyoxal consistently formed films more slowly whenever there was a detectable difference in rates (see Fig. S6 in the Supplement). Third, films formed both in the dark and under fluorescent light with no consistent trend in formation rate. Finally, films formed days to months more slowly at colder temperatures but, importantly for application to the cold UT/LS, were eventually observed (after approximately 100 days) even at the lowest temperature (−19°C) examined.

### 3.2 Chemical composition of surface films

The highly colored nature of the surface films (only formed on solutions containing propanal) is strong evidence for aldol condensation products, as aldol condensation is the only potential reaction (Fig. 1) of propanal in sulfuric acid that can result in products with the conjugation required to cause absorption of visible light. In fact, multiple aldol condensation steps are required to produce sufficient conjugation, since the first aldol condensation product of propanal (2-methyl-2-pentenal, see Fig. 1a) is colorless with λ_max_ for the *π* → π* transition of ∼266 and ∼233 nm in 75 wt % H_2_SO_4_ (Casale et al., 2007) and water (our standard), respectively. Further conjugation from additional aldol condensation reactions of 2-methyl-2-pentenal with propanal or with itself is required to shift absorption into the visible. Although products from multiple aldol condensation steps are almost certainly responsible for the film color, these chromophores are not necessarily the major chemical components of the films, so ATRFTIR and ^1^H NMR spectroscopies were used to analyze the chemical composition of the surface films. The combined results of these two techniques provide evidence that the films are a mixture of aldol condensation products (mainly 2-methyl-2-pentenal and 1,3,5-trimethylbenzene) and acetals (mainly 2,4,6-triethyl-1,3,5-trioxane and longer-chain linear polyacetals) as detailed in Sect 3.2.1 through 3.2.3 below. The detailed chemical analysis in these sections is presented for surface films formed on 0.30 M propanal/48 wt % H_2_SO_4_ as a starting point, since surface films were only formed on solutions containing propanal and propanal formed films fastest at 48 wt % H_2_SO_4_. These films were stored at room temperature under constant fluorescent light and were sampled and analyzed 7 days after mixing the solutions. Section 3.3–3.5 subsequently address the impact of varying the temperature, illumination, organic concentration, film age, acidity and organic mixture from this base case.

#### 3.2.1 Aldol condensation products

**Figure 2:**
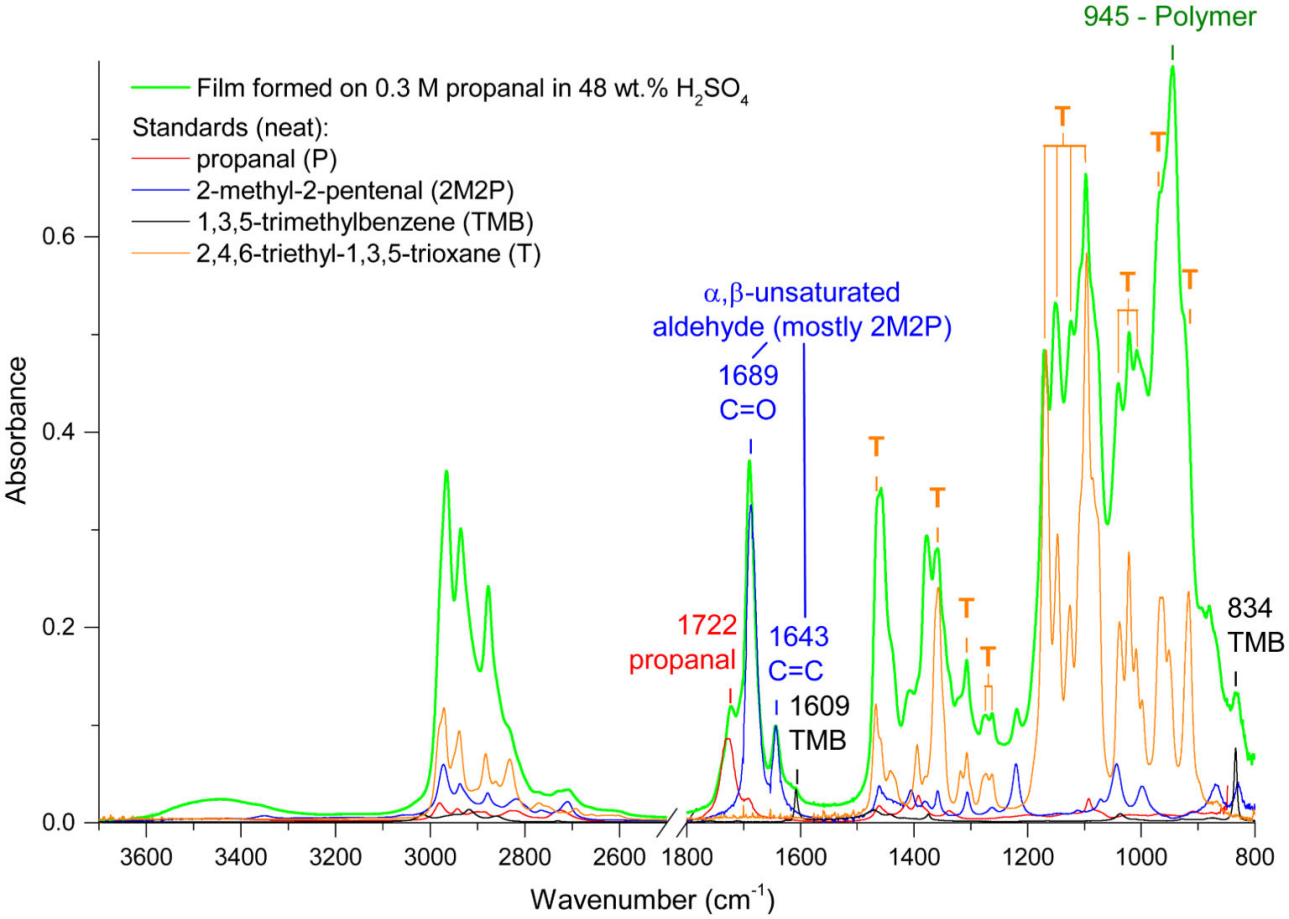
Typical ATR-FTIR spectrum of a surface film formed on 0.30 M propanal in 48 wt % H_2_SO_4_ (7 days after mixing) compared to neat standards. Spectra of standards for propanal, 2-methyl-2-pentenal, 1,3,5-trimethylbenzene and 2,4,6-triethyl-1,3,5-trioxane are scaled to indicate their maximum possible contribution to the film spectrum. The positions of the major trioxane peaks are indicated with the abbreviation T to illustrate their presence in the spectrum of the film. Other important peaks are labeled with wave number and their assignments as discussed in the text. Note that the region from 2500 to 1800 cm^−1^ lacks peaks and is omitted for clarity. For details of the lower-intensity traces see Fig. S2 in the Supplement, which provides a version of this figure covering the smaller absorbance range of 0–0.15.

Figure 2 presents a typical ATR-FTIR spectrum of a surface film formed on a 7-day-old 0.30 M propanal/48 wt % H_2_SO_4_ mixture (in green) along with spectra of four standards for comparison. The strong absorption band in the film spectrum at 1689 cm^−1^ and the band at 1643 cm^−1^ are consistent with the characteristic C = O and C = C stretching vibrations, respectively, of an *α*, *β*-unsaturated aldehyde which is produced by aldol condensation (Fig. 1a). The spectrum of neat 2-methyl-2-pentenal shown in Fig. 2 (blue) displays these bands at 1687 and 1643 cm^−1^ and is scaled to illustrate the maximum amount of the film spectrum that could be explained by its presence (limited by the size of the C = C band at 1643 cm^−1^). An additional C = O peak at 1722 cm^−1^ occurs in the saturated aldehyde stretching region and is assigned to unreacted propanal. In Fig. 2, the spectrum of neat propanal (red) is also scaled to illustrate its potential contribution to the spectrum of the film.

**Figure 3:**
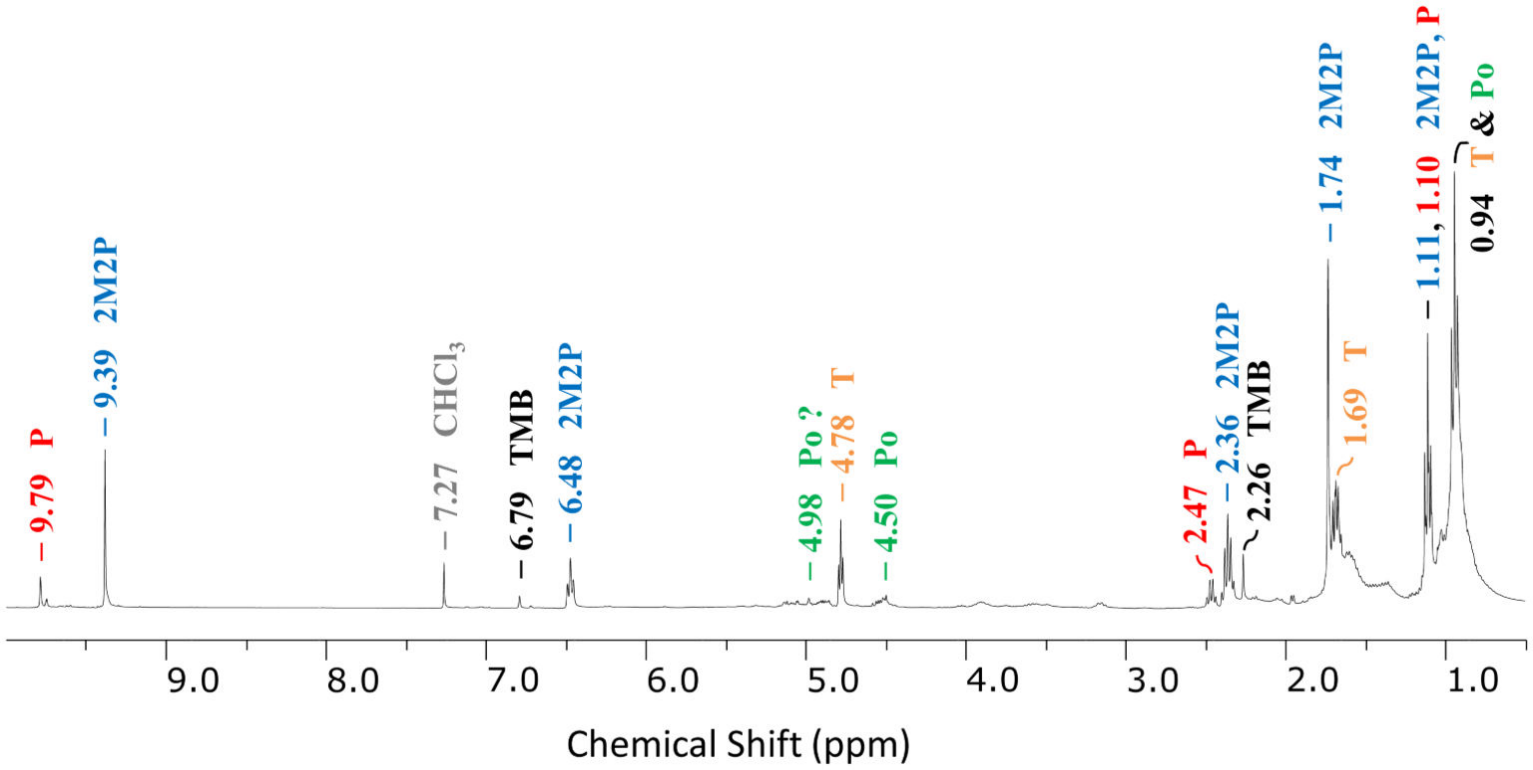
^1^H NMR spectrum of a surface film formed on 0.30 M propanal in 48 wt % H_2_SO_4_ (7 days after mixing). The film was dissolved in CDCl_3_. The ATR-FTIR spectrum for this same film is shown in Fig. 2. All major peaks have been assigned to the following five dominant species: P = propanal, 2M2P =2-methyl-2-pentenal, TMB =1,3,5-trimethylbenzene, Po =polymer, T =2,4,6-triethyl-1,3,5-trioxane.

The ^1^H NMR spectrum for this film presented in Fig. 3 indicates that 2-methyl-2-pentenal is the dominant species since it contains strong peaks (assigned in Fig. 3) corresponding to all five types of hydrogens in 2-methyl-2-pentenal in the correct multiplicity and within 0.03 ppm of our standard. Although some of the peaks are too small or too close to interfering peaks to integrate reliably, the relative peak intensities are also roughly consistent with the standard. Residual propanal is similarly positively identified by comparison to the standard as shown by peak assignments in Fig. 3. There are no additional detectable NMR peaks consistent with linear compounds with additional units of conjugation due to multiple aldol condensation steps, indicating that they must be significantly less abundant than 2-methyl-2-pentenal and therefore will not contribute substantially to the FTIR spectrum either. For example, the protons labeled A and B in 2,4-dimethyl-2,4-heptadienal (Fig. 1) would be expected to appear as singlets with chemical shifts near those for 2,4-hexadienal (Spectral Database for Organic Compounds, SDBS, 2014) at 9.5 and 7.1 ppm, respectively.

Although there is no NMR evidence for linear aldol condensation products beyond 2-methyl-2-pentenal, NMR peaks at 2.26 and 6.79 ppm confirm the presence of 1,3,5-trimethylbenzene (mesitylene) (SDBS), which has previously been observed to form in reactions of propanal over acidic zeolite catalysts (Hoang et al., 2010). Hoang et al. (2010) proposed that 1,3,5-trimethylbenzene was formed by acid-catalyzed cyclization and subsequent dehydration of the trimer formed by aldol condensation (2,4-dimethyl-2,4-heptadienal) of propanal as shown in Fig. 1b, which is a reasonable mechanism for sulfuric acid solutions as well. Furthermore, the trimer formed by aldol condensation of acetone has also been shown to cyclize to form 1,3,5-trimethylbenzene in sulfuric acid (Duncan et al., 1998; Kane et al., 1999; Klassen et al., 1999). In Fig. 2, the ATR-FTIR spectrum of neat 1,3,5-trimethylbenzene (black) is scaled to the film spectrum to indicate its maximum potential contribution. The comparison shows that the film spectrum is consistent with the presence of some 1,3,5-trimethylbenzene in the film since it has bands corresponding to the two most intense 1,3,5-trimethylbenzene bands at 834 and 1609 cm^−1^, the latter of which lies in the region for aromatic skeletal vibrations and, therefore, cannot be explained by any other potential products.

Although 2-methyl-2-pentenal and 1,3,5-trimethylbenzene are shown here to be the major products resulting from aldol reactions, both are colorless, so the more highly conjugated compounds formed by further aldol condensation steps that are presumably responsible for the film color must be minor constituents. Therefore, there must also be additional compounds present in the film to explain the strength of the peaks that appear in the FTIR spectrum between 1500–800 and 3000–2800 cm^−1^.

#### 3.2.2 Ethers: acetals/hemiacetals and linear/cyclic polyacetals

In addition to aldol condensation products, the FTIR and NMR spectra both also display evidence for ether groups (CO-C) due to strong peaks in the 1200–1000 cm^−1^ region of the FTIR spectrum (Fig. 2) and peaks in the 4.5–5.1 ppm region of the NMR spectrum. Species that could be responsible for these ether signatures include hemiacetals, acetals and/or higher-order polyacetal polymers which can form from the reaction of propanal with one or more of its hydrates (diols) (Fig. 1c) or from cyclotrimerization of propanal to form the cyclic acetal, 2,4,6-triethyl-1,3,5-trioxane (Fig. 1d). Of these potential products, the cyclotrimer is most easily confirmed since it is readily identified by comparison of the FTIR and NMR spectra of the film to the spectra of 2,4,6-triethyl-1,3,5-trioxane as indicated by the peaks assigned to the trioxane (T) in Figs. 2 and 3. Specifically, the ^1^H NMR spectrum of the film contains all three of the peaks in the reference spectrum (SDBS): a triplet at 4.78 ppm, a complex multiplet at 1.67 ppm and a triplet at 0.94 ppm (although the broad peak group at 0.94 ppm can only be partially due to the trioxane due to its strong intensity relative to the other trioxane peaks). Similarly, as shown by assignments in Fig. 2, at least 13 peaks in the FTIR spectrum of the film correspond to peaks in the spectrum of neat 2,4,6-triethyl-1,3,5-trioxane (including all six of the strongest peaks between 1500 and 900 cm^−1^). Furthermore, previous studies of 2,4,6-triethyl-1,3,5-trioxane report that it phase separates upon formation from propanal/catalyst solutions (Sato et al., 1993, 1991), consistent with our surface film formation.

Upon assignment of the cyclotrimer peaks, only one major peak in the FTIR spectrum of the film remains unexplained by species identified thus far (2,4,6-triethyl-1,3,5-trioxane, 2-methyl-2-pentenal, 1,3,5-trimethylbenzene and propanal). This peak at 945 cm^−1^ is, however, the strongest peak in the spectrum of the film and therefore must be a major peak in the spectrum of the absorbing species. The hemiacetal and single acetal formed by propanal (Fig. 1c) are unlikely to be responsible for the peak at 945 cm^−1^ since they would be expected to produce their strongest bands at higher frequencies. Specifically, the hemiacetal would produce a strong FTIR absorption band in the 1150–1085 cm^−1^ region from the asymmetric stretch of its single ether group, while the acetal contains the C-O-C-O-C moiety which would produce five characteristic bands between 1200 and 1020 cm^−1^ (Bergmann and Pinchas, 1952). Furthermore, both the hemiacetal and acetal are also unlikely to be major ether constituents since only a weak peak exists in the OH stretching region (3500–3400 cm^−1^) where a stronger peak (with respect to the peaks in the ether region) would be expected due to the OH groups.

Instead, the strong peak at 945 cm^−1^ most likely results from longer-chain polymers of propanal (polyacetal in Fig. 1c) due to C-O-C-O-C stretching bands that are shifted to lower frequencies with the addition of additional ether groups. Spectra of polymers of various small aldehydes (formaldehyde, acetaldehyde and propanal) which contain the same polymethoxy (-C-O-)*_n_* backbone display only very weak OH stretching bands but multiple very strong, broad, overlapping absorption bands between 925 and 975 cm^−1^ (Novak and Whalley, 1959a, b, 1962; Vogl, 1964a, b). Although the peak at 945 cm^−1^ does not exactly match any of the three strongest peaks (975, 960 and 925 cm^−1^) in this region in the Novak and Whalley (1959a) spectrum of the polymethoxy polymer formed by pressurization of propanal, there is such broad absorption in the entire 980–920 cm^−1^ region of the polymer spectrum that a peak near 945 cm^−1^ may not be distinguishable. Furthermore, the polymer present in our surface film is likely to display different relative intensities of the peaks in this region due to differences in degree of polymerization and/or differences in relative quantities of rotational isomers (Novak and Whalley, 1959a). Additionally, bands may also be shifted in frequency due to different interactions between polymer chains (Novak and Whalley, 1959a) in the complex surface film matrix. Finally, the NMR spectrum of the film is also consistent with the presence of propanal polymer since the 4.5–5.1 ppm region contains multiple unassigned peaks consistent with ethers and similar to the broad group of unresolved peaks from 4.5 to 5.0 ppm that characterizes the NMR spectrum of the polymethoxy polymer formed by acetaldehyde (Vogl, 1964a), while CH_2_ and CH_3_ protons from the ethyl chains are likely responsible for peaks in the 1.0–1.7 ppm region and for a portion of the triplet at 0.94 ppm, respectively.

After this identification of polymers of propanal, we note that all of the major bands in the infrared spectrum that could not be explained by aldol condensation products either correspond to 2,4,6-triethyl-1,3,5-trioxane or could reasonably be assigned to longer-chain linear propanal polymers.

#### 3.2.3 Other potential film components: organosulfates and minor species

**Figure 4:**
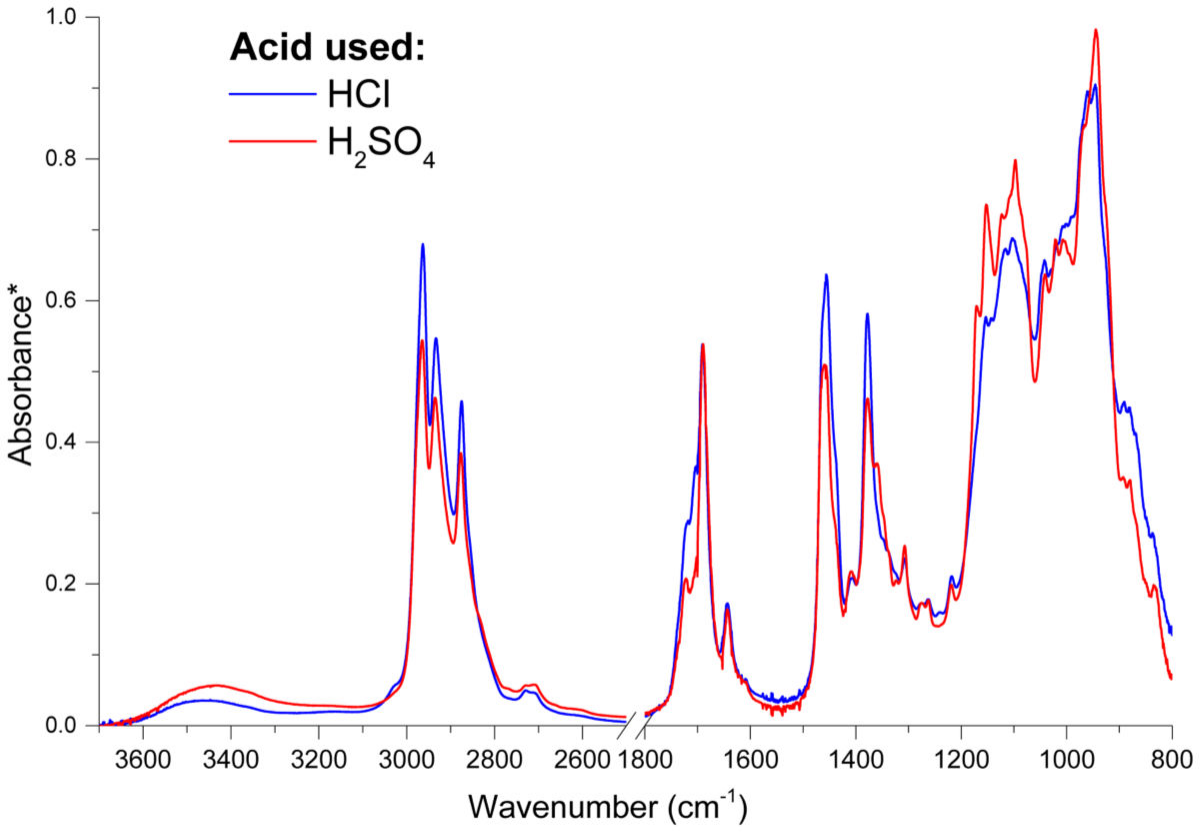
ATR-FTIR spectra of films formed on 0.30 M propanal at pH = −0.85 in H_2_SO_4_ (48 wt %) and in HCl (7 days after mixing). The region from 2500 to 1800 cm^−1^ is omitted for clarity. *Absorbance spectra are scaled to the C=O peak at 1690 cm^−1^ from aldol condensation products (predominantly 2-methyl-2-pentenal).

In order to test for the presence of organosulfates, reaction mixtures were prepared with hydrochloric acid at the same pH as the sulfuric acid mixtures. The formation of surface films on these mixtures demonstrates that organosulfates are not necessary for film formation, and the similarity of the ATR-FTIR spectra for films formed on sulfuric acid and hydrochloric acid solutions (example shown in Fig. 4 for 0.30 M propanal/48 wt % H_2_SO_4_) demonstrates that organosulfates are not present in significant quantities. We note, however, that organosulfates could still be produced in the sulfuric acid solutions, where they would be expected to remain due to their ionizability.

Although all of the major peaks (and many of the minor peaks) in both the FTIR and NMR spectra of the film can be assigned to the chemical species discussed thus far, some small unassigned peaks (e.g., NMR peaks at 3.2 and 3.9 ppm) indicate the presence of other minor species. These could include products of multiple aldol condensation steps, aldols that have not lost water through the condensation process (see Fig. 1), the hemiacetal and acetal formed by propanal, other acetals that could also potentially be formed by reactions of aldol condensation products with propanal and/or products from oxidation of films by light/air.

### 3.3 Effects of light exposure, temperature, propanal concentration and film age

The preceding detailed chemical analyses were presented for the base case of a film formed on a 7-day-old solution of 0.30 M propanal in 48 wt % sulfuric acid, stored at room temperature under fluorescent room light. Very similar NMR spectra were obtained from films formed on solutions that were stored under different conditions (dark and/or 0°C), that were younger (1 and 4 days) and older (68 and 134 days) or that were formed at lower propanal concentration (0.030 M); these spectra confirm the presence of the same major chemical species. Spectra of films formed in the dark are not detectably different than those formed in the light, but films formed at different ages, at 0 °C or at lower propanal concentration display the following significant differences in relative peak areas between chemical species when compared to the base case.

There were two detectable trends in NMR peak area ratios with film age. First, the trioxane (4.78 ppm) peak area decreased with age relative to all other species that produced peaks separated well enough for integration (2-methyl-2-pentenal (9.39 ppm), trimethylbenzene (6.79 ppm) and propanal (9.79 ppm)); furthermore, the oldest samples (68 and 134 days) lacked any detectable trioxane. Trioxane peaks also decreased relative to the peaks in the ATR-FTIR spectra assigned to long-chain polymers. Therefore, since trioxane decreases with time relative to all other major film species and the films grow thicker with time, it is possible that trioxane is initially formed rapidly, followed by slower formation of all other film species. Second, the trimethylbenzene (6.79 ppm) to 2-methyl-2-pentenal (9.39 ppm) peak area ratio increased with age (by a factor of 2 to 3 going from 1–7-day-old samples to 68- and 134-day-old samples). Since 2-methyl-2-pentenal is a precursor for trimethylbenzene formation, this result suggests that trimethylbenzene formation continues beyond 1 week.

Although solutions stored at the lowest temperature of −19°C did not produce sufficient quantities of film for analysis, the NMR spectrum of a 73-day-old film formed at 0 °C showed higher relative levels of trioxane and lower relative levels of trimethylbenzene than those formed at room temperature. This result is consistent with reactions that proceed more slowly at lower temperature, according to the previously noted trends with age.

Finally, solutions with lower propanal concentration (0.030 vs. 0.30 M) did not produce a sufficient quantity of film for reliable removal and spectral analysis without contamination by the underlying sulfuric acid solution. However, one weak NMR spectrum of a 16-day-old sample was obtained that allows positive detection of trimethylbenzene and 2-methyl-2-pentenal and indicates likely presence of long-chain polymers due to multiple overlapping peaks similar to those previously assigned to protons on the polymer ethyl chains (1.0–1.7 and ∼0.94 ppm). Trioxane could not be detected above the noise; however, we note that low trioxane content could be due to the older film age since the trimethylbenzene to 2-methyl-2-pentenal ratio is high and therefore also consistent with older films formed on 0.30 M propanal solutions.

### 3.4 Effect of acidity

**Figure 5:**
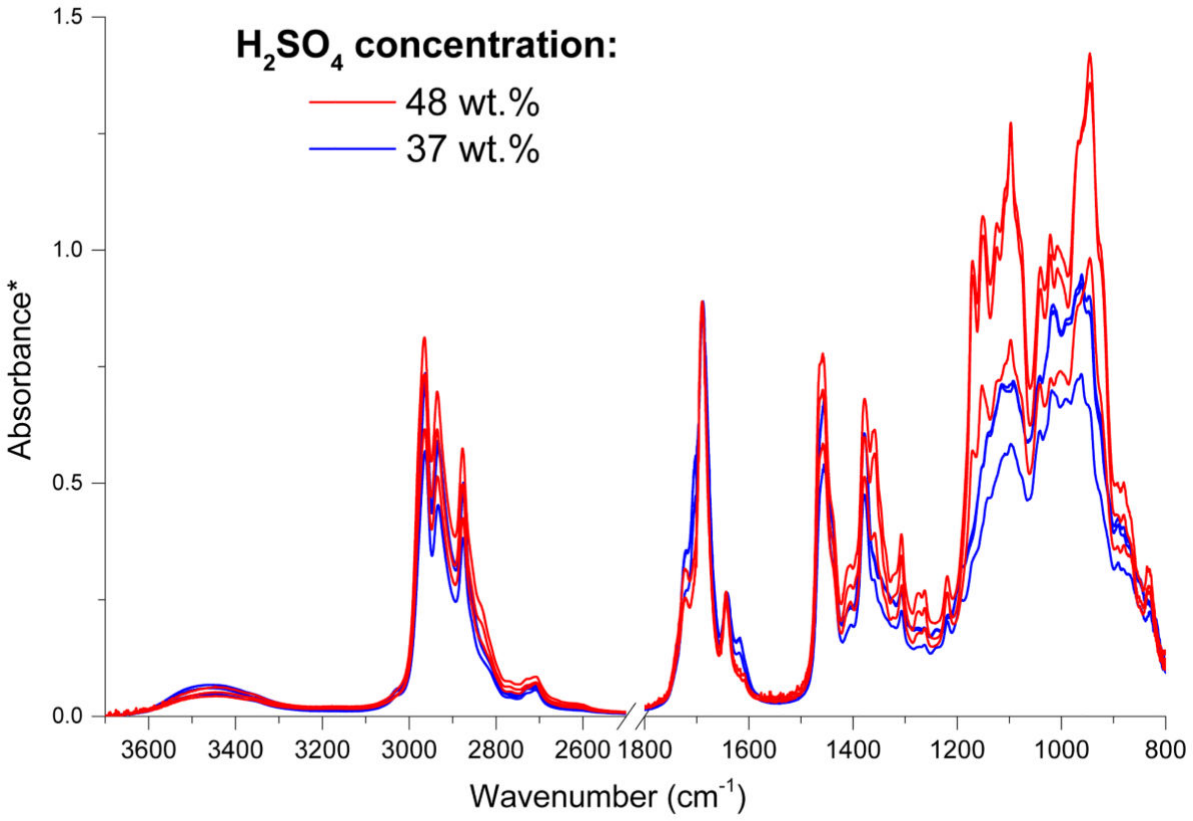
Effect of acidity on the ATR-FTIR spectra of surface films formed on 0.30 M propanal in H_2_SO_4_ (7 days after mixing). Trip-licates are shown for both 48 and 37 wt % H_2_SO_4_. The region from 2500 to 1800 cm^−1^ is omitted for clarity. *Absorbance spectra are scaled to the C=O peak at 1690 cm^−1^ from aldol condensation products (predominantly 2-methyl-2-pentenal) in order to illustrate differences between relative peak intensities.

As discussed in Sect. 3.1, acidity has a complex effect on the formation rates of the surface films that varies depending on the organic mixture. In general, films tended to form faster as the acidity increased from 19 to 37 to 48 wt % H_2_SO_4_, but films formed more slowly or not at all at the highest acidity (76 wt % H_2_SO_4_) in all mixtures except propanal/glyoxal. The FTIR spectra of films formed on mixtures of 0.30 M propanal in 48 and 37 wt % H_2_SO_4_ solutions in Fig. 5 (shown in triplicate) demonstrate that there are also chemical differences in films formed at different acidities. The spectra are scaled to the C=O peak at 1690 cm^−1^ from aldol condensation products (predominantly 2-methyl-2-pentenal) in order to illustrate differences in relative peak intensities. Although there is considerable variability in relative peak intensities among the spectra of replicates (most likely due to film in-homogeneity), the peaks in the 1200–900 cm^−1^ region are generally larger relative to the C=O peak at the higher acidity (red), indicating a larger relative contribution to the film from the 2,4,6-triethyl-1,3,5-trioxane and longer-chain polyacetal polymers that absorb in this region. In addition, the films formed at the higher acidity also have smaller peaks at 1608 cm^−1^, indicating smaller concentrations of 1,3,5-trimethylbenzene relative to aldol condensation products. Both of these trends are confirmed by NMR spectroscopy (data not shown). The presence of more 2,4,6-triethyl-1,3,5-trioxane and polymers at higher acidities is consistent with faster film formation at higher acidities in the 19–48 wt % H_2_SO_4_ range since these species are most likely responsible for the phase separation into a surface film. Additionally, slower film formation at the highest acidity (76 wt %) is potentially due to low water content that reduces the formation of the diols required to begin the polymerization process (see Fig. 1c).

### 3.5 Cross-reactions with glyoxal and methylglyoxal

**Figure 6:**
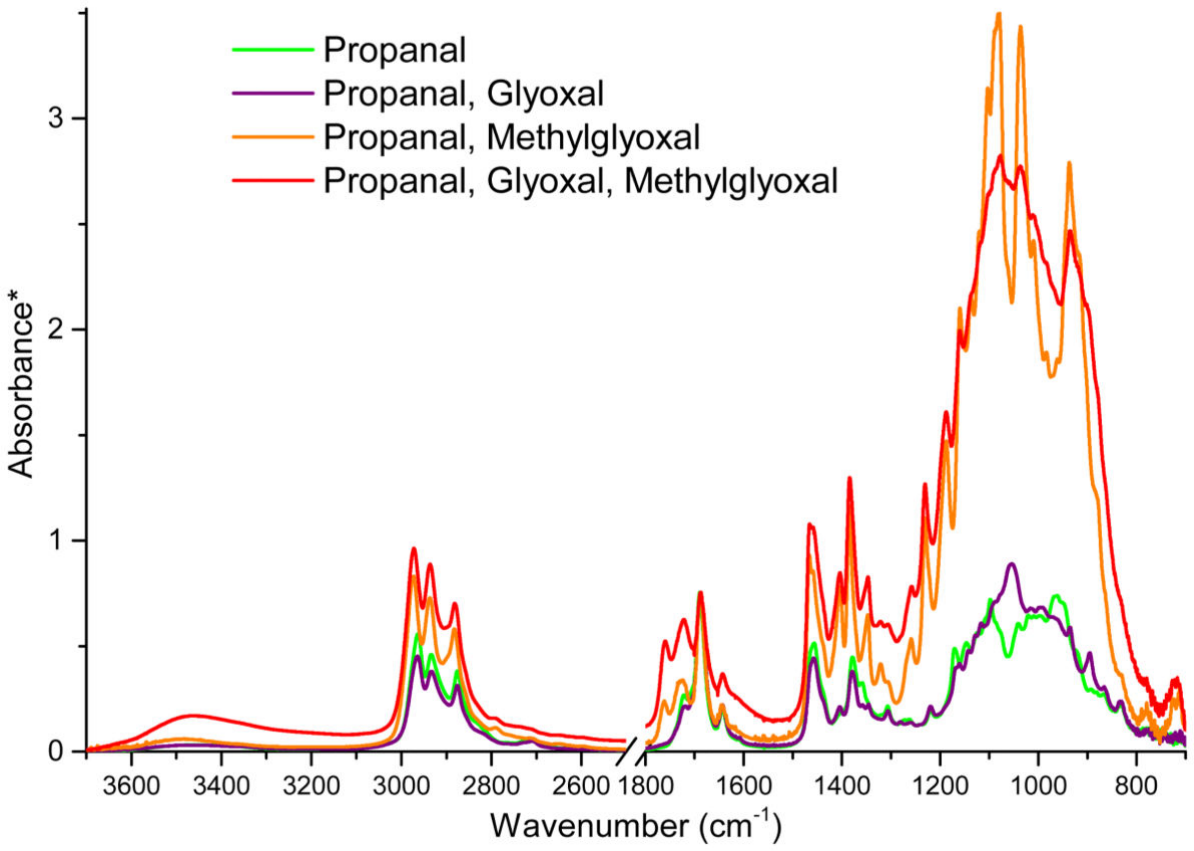
ATR-FTIR spectra of surface films formed on mixtures of propanal with glyoxal and/or methylglyoxal in 48 wt % H_2_SO_4_ (7 days after mixing). Solutions are 0.30 M in each organic. The region from 2500 to 1800 cm^−1^ is omitted for clarity. *Absorbance spectra are scaled to the C=O peak at 1690 cm^−1^ from aldol condensation products (predominantly 2-methyl-2-pentenal) in order to illustrate differences between relative peak intensities. Spectra of replicates are provided in the Supplement (Figs. S3 and S4).

To examine the potential effect of additional organic species with carbonyl groups on the formation of films, mixtures of propanal with glyoxal and/or methylglyoxal were also examined. Although glyoxal and methylglyoxal did not form films in the absence of propanal, the mixtures of 0.03 M propanal and 0.03 M glyoxal formed films faster than 0.03 M propanal alone suggesting that products of cross-reactions between glyoxal and propanal participate in film formation, resulting in faster film formation due to higher total concentrations of reactants available for film-forming reactions. In contrast, mixtures of 0.03 M propanal and 0.03 M methylglyoxal formed films more slowly than 0.03 M propanal alone. A comparison of FTIR spectra of films formed on various organic mixtures in 48 wt % H_2_SO_4_ all prepared on the same day are shown in Fig. 6. Because significant variability in relative peak intensities exists in replicate FTIR spectra of the films most likely due to inhomogeneity in the solid mixtures of multiple chemical species, a complete set of replicate spectra are provided in the Supplement (Figs. S3 and S4) to demonstrate that the differences between organic mixtures discussed here are in fact due to differing chemical pathways and are not simply sampling artifacts. The spectra in Fig. 6 are again scaled to the C=O peak at 1690 cm^−1^ from propanal aldol condensation products in order to illustrate differences between relative peak intensities. The spectra of the films from propanal and propanal/glyoxal are nearly identical in the 1800–1600 cm^−1^ region, indicating that both films include 2-methyl-2-pentenal and unreacted propanal in similar ratios. Conversely, the spectral pattern in the 1200–900 cm^−1^ region for the propanal/glyoxal film does not correspond to the spectrum of 2,4,6-triethyl-1,3,5-trioxane as it does for the propanal-only film. Since glyoxal did not form films by itself, this infrared signature is most likely due to products of cross-reactions between propanal and glyoxal.

The FTIR spectrum of the film from propanal and methylglyoxal deviates even farther from that of only propanal. Not only does it lack the signature of 2,4,6-triethyl-1,3,5-trioxane, indicating products of cross-reactions, but, additionally, absorbance in the entire 1500–900 cm^−1^ region is much stronger relative to the aldol condensation peak at 1690 cm^−1^, indicating a stronger relative contribution from acetal species. Finally, it is intriguing that (1) the spectrum of the film formed on the propanal/glyoxal/methylglyoxal mixture is quite similar to that for the propanal/methylglyoxal mixture, differing only in relative peak ratios (a result that remained true even when the methylglyoxal concentration was reduced by up to a factor of 10 but not by a factor of 100); and (2) that the rate of film formation was decreased from the rate for the propanal/glyoxal mixture. This could indicate that glyoxal is somehow inhibited from participating in film-forming reactions by the presence of methylglyoxal. The mechanism for such inhibition is unclear, but plausible explanations include cross-reactions of glyoxal with methylglyoxal that are faster than those with propanal but that do not result in products that partition to the film and/or dimerization reactions of propanal with methylglyoxal that are faster than those with glyoxal but that subsequently require more time to form polymers that are large enough to partition into the film.

### 3.6 UV–visible spectra of solutions

**Figure 7:**
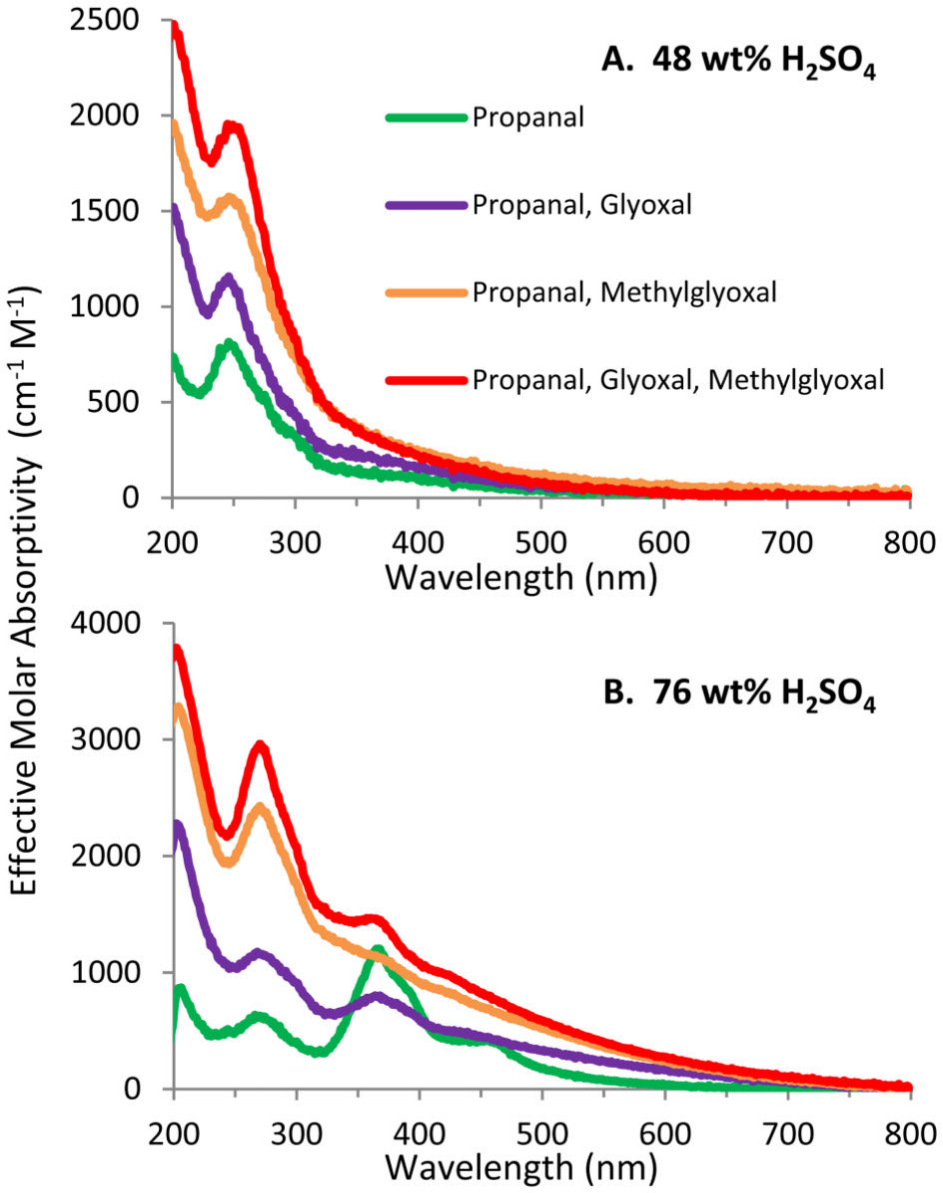
UV–visible absorption spectra of aged film-forming solutions. Solutions are 0.030 M in each organic and were prepared in (a) 48 wt % H_2_SO_4_ or (b) 76 wt % H_2_SO_4_ and stored for 274 days. “Effective” molar absorptivity is calculated based only on the concentration of the propanal reactant (0.030 M) so that any changes in absorbance (compared to the propanal-only spectrum) must be due to the presence of the additional organic species.

Although the focus of this work is on characterization of the surface films, the UV–visible absorption spectra of aged organic/sulfuric acid solutions were also examined in order to give some insight into the potential formation of highly absorbing species over the long residence time of lower stratospheric aerosols (∼2 years). Figure 7a (green) shows that a solution of 0.030 M propanal in 48 wt % sulfuric acid allowed to age for 274 days had two strong absorption peaks around 200 and 245 nm, most likely corresponding to species also observed in the films: 1,3,5-trimethylbenzene and 2-methyl-2-pentenal, which absorb in water at ∼200 and 234 nm, respectively. More importantly, the absorbance extends significantly into the visible. There are no other distinguishable peaks, but the absorbance is most likely due to overlapping peaks from various longer oligomers formed by additional aldol condensation reactions of 2-methyl-2-pentenal with propanal and/or with itself. Each sequential aldol condensation step would add another unit(s) of conjugation and thereby shift the absorption peak to longer wavelengths. This interpretation is supported by the observation that when the acidity was increased to 76 wt % sulfuric acid (Fig. 7b), the intensity of the peak corresponding to 2-methyl-2-pentenal was reduced (or even absent) and additional peaks became distinguishable at longer wavelengths (270, 365, 388 (shoulder) and 458 nm). Nozière and Esteve (2007) observed a similar spectrum for reaction products of propanal in 96 wt % sulfuric acid and also ascribe these long wavelength peaks to oligomers from aldol condensation reactions. Although they suggest that the peak in their spectrum near 270 nm may be propanal itself, this cannot be the case for our samples since the molar absorptivity of propanal is too small at ∼9 cm^−1^ M^−1^ (Xu et al., 1993).

The absorption spectra of mixtures of propanal with glyoxal and/or methylglyoxal are also presented in Fig. 7. “Effective” molar absorptivities are calculated based only on the concentration of the propanal reactant (0.030 M) so that any changes in absorbance (compared to the propanal-only spectrum) must be due to the presence of the additional organic species. At both acidities, absorbance in most of the spectrum is increased, with methylglyoxal having a larger effect than glyoxal, suggesting that the added organic species are undergoing aldol condensation either via reactions with propanal or self-reactions. Although some of the additional absorption may be due to glyoxal and methylglyoxal themselves, molar absorptivities of these species are too small (Horowitz et al., 2001; Malik and Joens, 2000; Plum et al., 1983) to contribute significantly at least below 350 nm.

## 4 Discussion and atmospheric implications

The major species present in surface films formed on bulk solutions of propanal in sulfuric acid were identified as aldol condensation products (mainly 2-methyl-2-pentenal and 1,3,5-trimethylbenzene) and polyacetals (mainly 2,4,6-triethyl-1,3,5-trioxane and longer-chain linear polyacetals). Of these products, the polyacetal species (both cyclic and linear) are most likely to be primarily responsible for the separation of the organic species from the solution into a separate solid organic phase on the surface of the liquid due to their high molecular weight and higher hydrophobicity compared to the two observed aldol condensation products. Since the solid material in the laboratory samples rises to the surface of the solution due, at least in part, to its low density relative to sulfuric acid, it is unclear whether similar insoluble acetals potentially formed from reactions of aldehydes in liquid UT/LS aerosols would exist as solid inclusions or as surface coatings (full or partial); the latter of which would be more likely to alter aerosol optical, chemical and/or cloud-nucleating properties.

Neither the solubility nor the reactive uptake coefficient of propanal in sulfuric acid has been measured, but, based on the low concentration of propanal vapor in the UT/LS (∼15 ppt at 11 km and presumably much lower in the stratosphere; Singh et al., 2004) and on the short lifetimes of gas phase aldehydes with respect to photolysis, uptake and reaction of propanal alone to form polyacetals is not expected to be a significant source of organic material in UT/LS aerosols. However, polyacetal formation from aldehydes in general could be important for three reasons. First, polyacetals may be formed from a variety of organic species since they have been observed to form from many aliphatic aldehydes (Vogl, 2000) and have specifically been observed in sulfuric acid for formaldehyde uptake (Iraci and Tolbert, 1997) and inferred for acetaldehyde uptake (Williams et al., 2010). Second, the rate of film formation was greatly enhanced by the presence of glyoxal, suggesting that carbonyl species already present in aerosols could enhance the reactive uptake and polyacetal formation of small aldehydes, consistent with previous experiments that demonstrated enhanced reactive up-take of acetaldehyde on sulfuric acid solutions containing formaldehyde (Williams et al., 2010) and enhanced reactive uptake of nonanal on mixed organic/sulfuric acid aerosols (Chan and Chan, 2011). Third, although aerosol concentrations of any one aldehyde are unlikely to result in significant self-polymerization, cross-reactions between aldehydes and/or between aldehydes and alcohols may be significant and are specifically shown here to occur between propanal and two common aerosol organic species (glyoxal and methylglyoxal).

Although uptake and dissolution of aldehydes onto sulfuric acid aerosols is the most likely method of polyacetal formation directly from small volatile mono-aldehydes like propanal, there may be more favorable methods for polyacetal formation in UT/LS aerosols. Since polyacetal formation requires multiple polymerization steps, the kinetics are likely to be greatly enhanced at higher concentrations of the organic reactants. One possibility for enhanced concentration of organic reactants is the potential preference of the reactants for the aerosol surface. If polyacetals partition to the aerosol surface as they do in our bulk experiments, their further polymerization with each other and with condensing organics would be enhanced; polymerization could be similarly enhanced if carbonyl and/or alcohol reactants partition to the aerosol surface or to organic inclusions. This possibility is supported by the recent work of Li et al. (2011), Schwier et al. (2010) and Sareen et al. (2010) demonstrating surface tension depression by surface-active species formed in solutions of formaldehyde, acetaldehyde, glyoxal and/or methylglyoxal in pure water and/or aqueous ammonium sulfate. Additionally, products of cross-reactions between methylglyoxal and formaldehyde or acetaldehyde had a larger effect on surface tension than could be explained by self-reactions alone.

An additional possibility for enhanced concentrations of organic reactants favorable for polyacetal formation is transport of organic-rich aerosols from the lower troposphere to the UT/LS. Polyacetal formation could be initiated on such aerosols upon condensation of H_2_SO_4_ and/or coagulation with H_2_SO_4_ particles formed near the tropopause. In order to evaluate the likelihood of this process, carbonyl species more typical of photochemically aged tropospheric aerosols (less volatile and likely more oxidized than propanal) should be evaluated for their potential to participate in acid-catalyzed polyacetal formation.

In addition to the major species identified in the films, aldol condensation products of higher order than the dimer, 2-methyl-2-pentenal, must also be present as minor species in order to account for the strong absorbance of visible light by the films. The absorbing species in the films most likely form in the solutions and then partition to the organic film since both the films and the solutions they form on are highly colored. If light-absorbing aldol condensation products in aerosols undergo similar partitioning into organic coatings, it would increase their potential impact on the optical properties of aerosols.

## 5 Conclusions

In summary, bulk solutions of propanal and sulfuric acid at UT/LS aerosol acidities produced surface films that absorbed strongly in the visible and that were composed primarily of aldol condensation products and polyacetals. When glyoxal and/or methylglyoxal were also present in solution, acetal products of cross-reactions were observed in the films while the presence of glyoxal also significantly increased the rate of film formation. Both of these results suggest that polyacetal reaction products such as those found in the films may be important when the variety of atmospheric gas and aerosol phase organic species available to serve as reactants is considered. Even if polyacetals and light-absorbing aldol condensation products do not account for a significant fraction of aerosol organic mass, their impact on aerosol radiative and CCN properties could be significant if they partition to the aerosol surface.

## Supplementary Material

Supplement
