# Supplementary material for: Complex chemical composition of colored surface films formed from reactions of propanal in sulfuric acid at upper troposphere/lower stratosphere aerosol acidities
Source: Atmos Chem Phys. Author manuscript; Available in PMC 2016 May 20. (PMC4874526; doi:10.5194/acp-15-4225-2015)
Supplement: Supplement [file NIHMS786039-supplement-Supplement.pdf]

Supplement of Atmos. Chem. Phys., 15, 4225–4239, 2015
http://www.atmos-chem-phys.net/15/4225/2015/
doi:10.5194/acp-15-4225-2015-supplement
© Author(s) 2015. CC Attribution 3.0 License.

Atmospheric Chemistry and Physics
Open Access

Supplement of

Complex chemical composition of colored surface films formed from reactions of propanal in sulfuric acid at upper troposphere/lower stratosphere aerosol acidities

A. L. Van Wyngarden et al.

Correspondence to: A. L. Van Wyngarden (annalise.vanwyngarden@sjsu.edu)

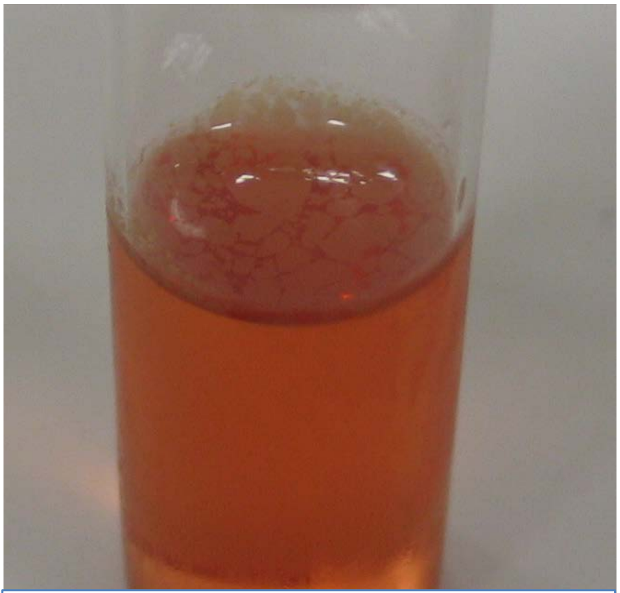

0.030 M propanal in 48 wt % sulfuric acid after 2.4 months

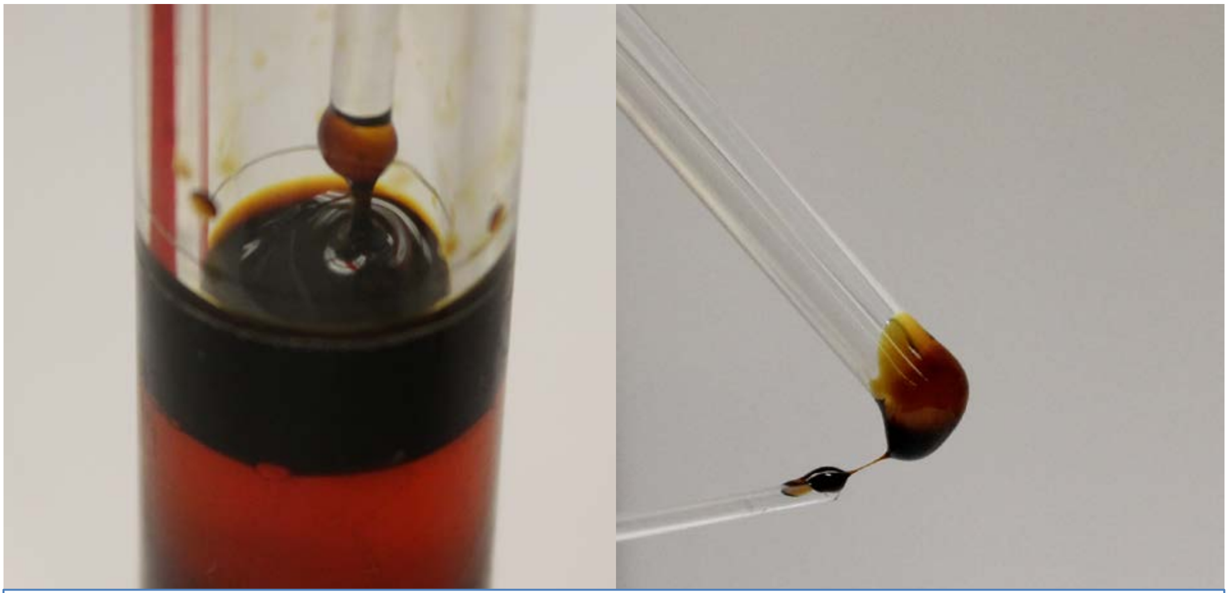

0.30 M propanal in 48 wt % sulfuric acid after 8.4 months

Figure S1. Photographs of sample surface films formed by propanal in 48 wt % sulfuric acid solutions. The photo on the top illustrates a surface film that has broken up (possibly due in part to movement of the vial) and no longer covers the entire liquid surface. The photos on the bottom show how the semi-solid film adheres to a stirring rod that has been touched to the surface (left) and some of the same film being removed from a stirring rod with a Pasteur pipette (right).

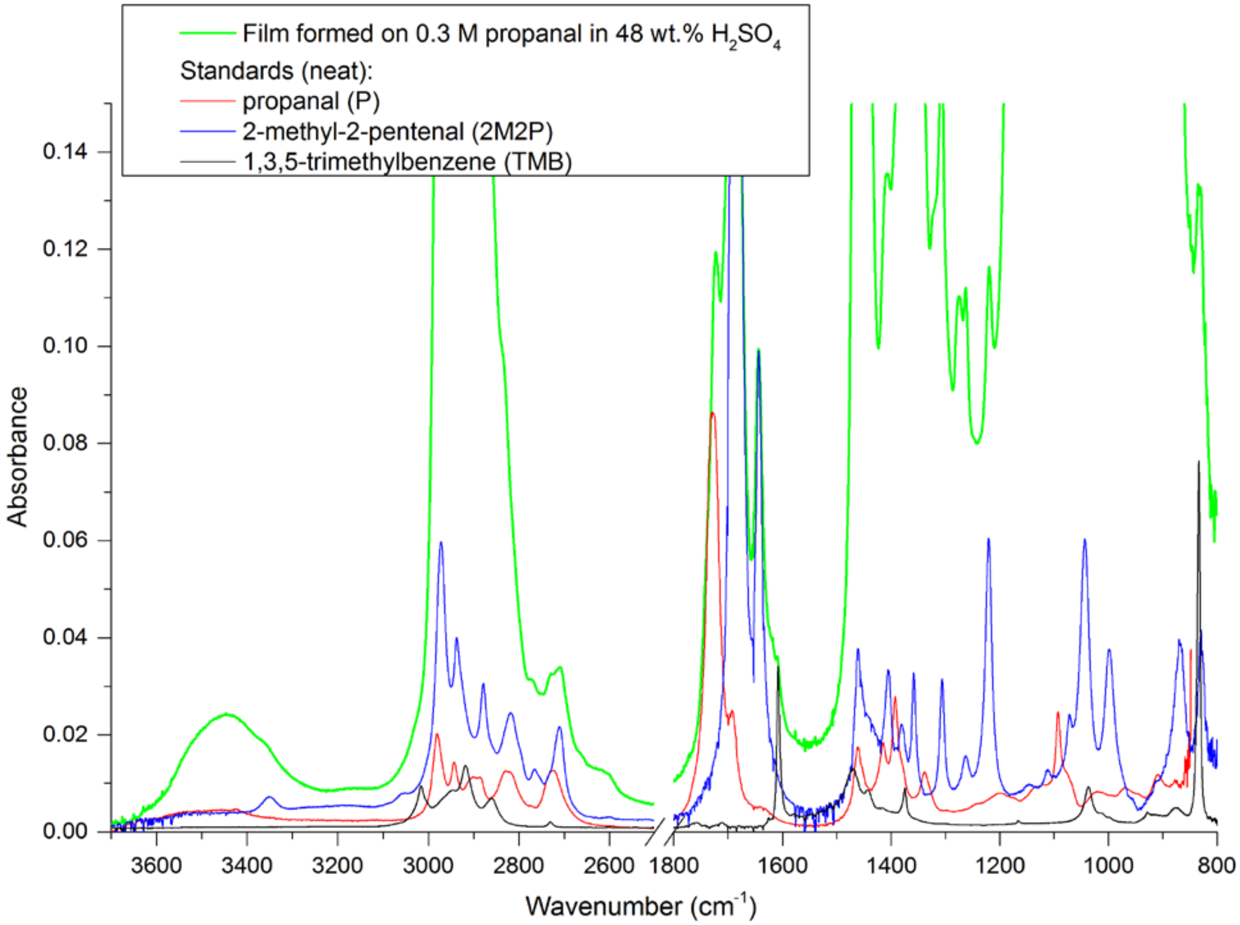

Figure S2. Details of the lower-intensity ATR-FTIR spectra in Figure 2 are shown using a smaller absorbance range. The spectrum of 2,4,6-triethyl-1,3,5-trioxane is not low intensity and is omitted for clarity.

Typical ATR-FTIR spectrum of a surface film formed on 0.30 M propanal in 48 wt % H_2SO_4 (7 days after mixing) compared to neat standards. Spectra of standards for propanal, 2-methyl-2-pentenal and 1,3,5-trimethylbenzene are scaled to indicate their maximum possible contribution to the film spectrum. Note that the region from 2500-1800 cm^{-1} lacks peaks and is omitted for clarity.

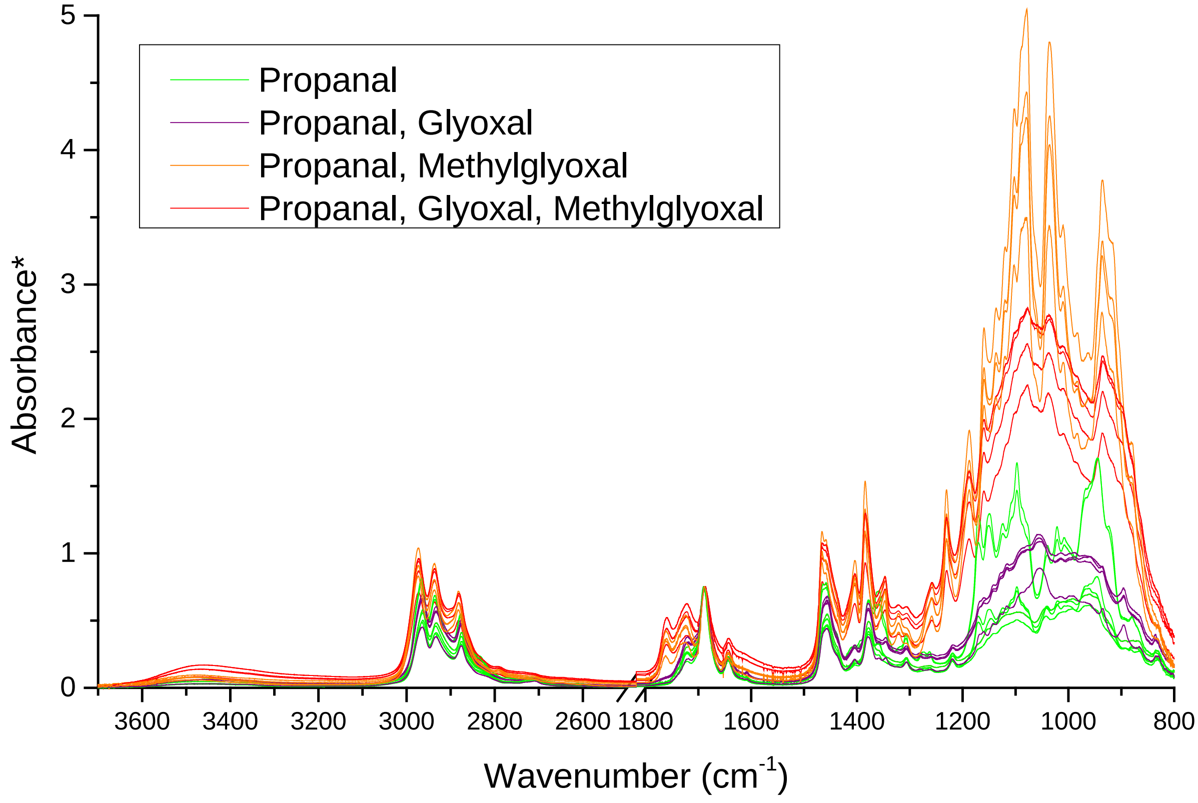

Figure S3. Replicates (corresponding to Figure 6) for the ATR-FTIR spectra of surface films formed on mixtures of propanal with glyoxal and/or methylglyoxal in 48 wt% H_2SO_4 (7 days after mixing). Solutions are 0.30 M in each organic. The region from 2500-1800 cm^{-1} is omitted for clarity. *Absorbance spectra are scaled to the C=O peak at 1690 cm^{-1} from aldol condensation products (predominantly 2-methyl-2-pentenal) in order to illustrate differences between relative peak intensities.

These replicates correspond to Figure 6 (where only one spectrum is shown per mixture for clarity) and show variability in relative peak intensities for spectra of films formed on replicate solutions of the same composition. This variability is most likely due to inhomogeneity in the solid mixtures of multiple chemical species. The replicate spectra are provided here to demonstrate that the differences between organic mixtures discussed in the manuscript are robust even when replicate variability is taken into account and are therefore due to differing chemical pathways and are not simply sampling artifacts. Figure S4 displays each organic mixture separately.

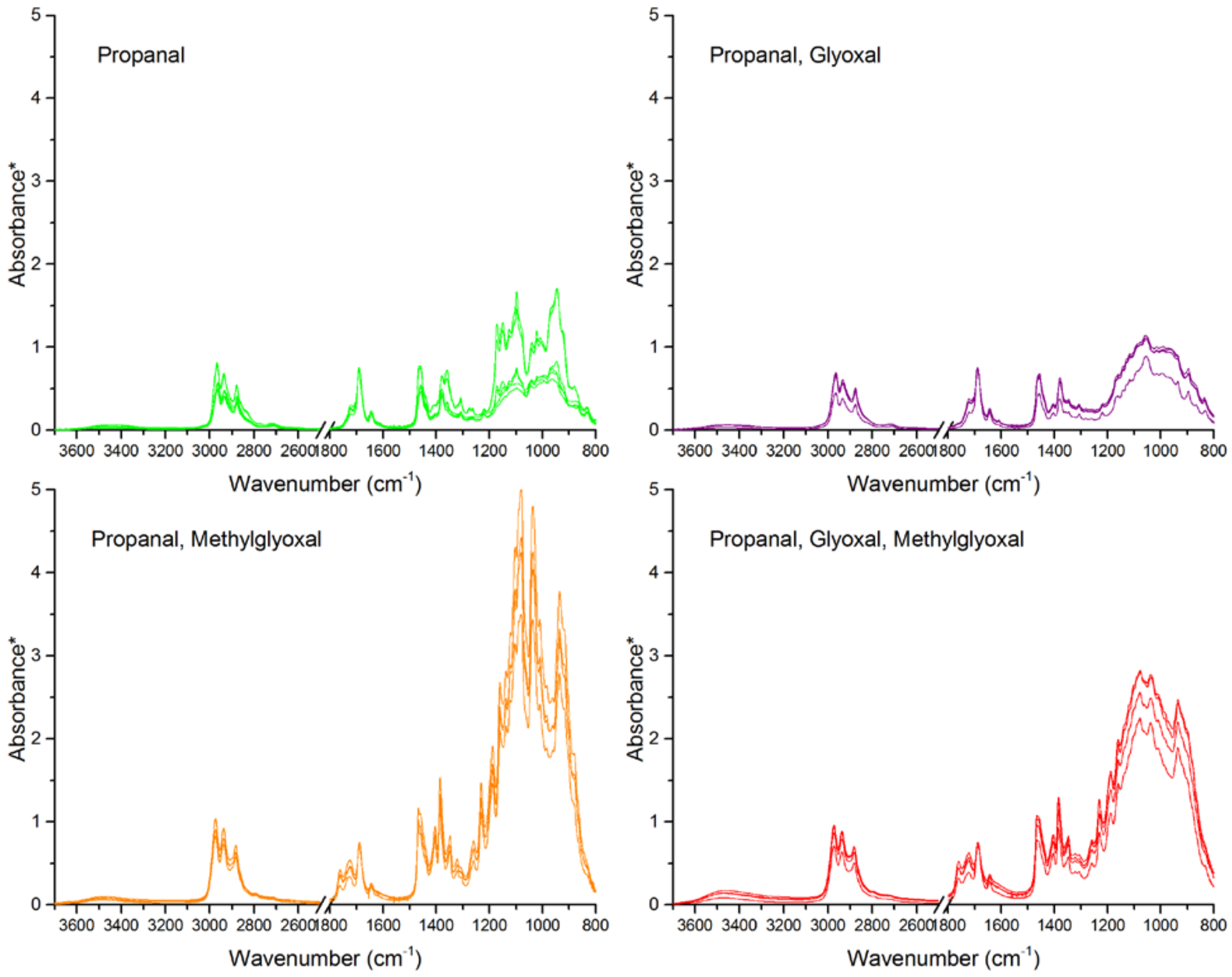

Figure S4. Figure S3 with each organic mixture displayed separately. See Figure S3 caption for complete description.

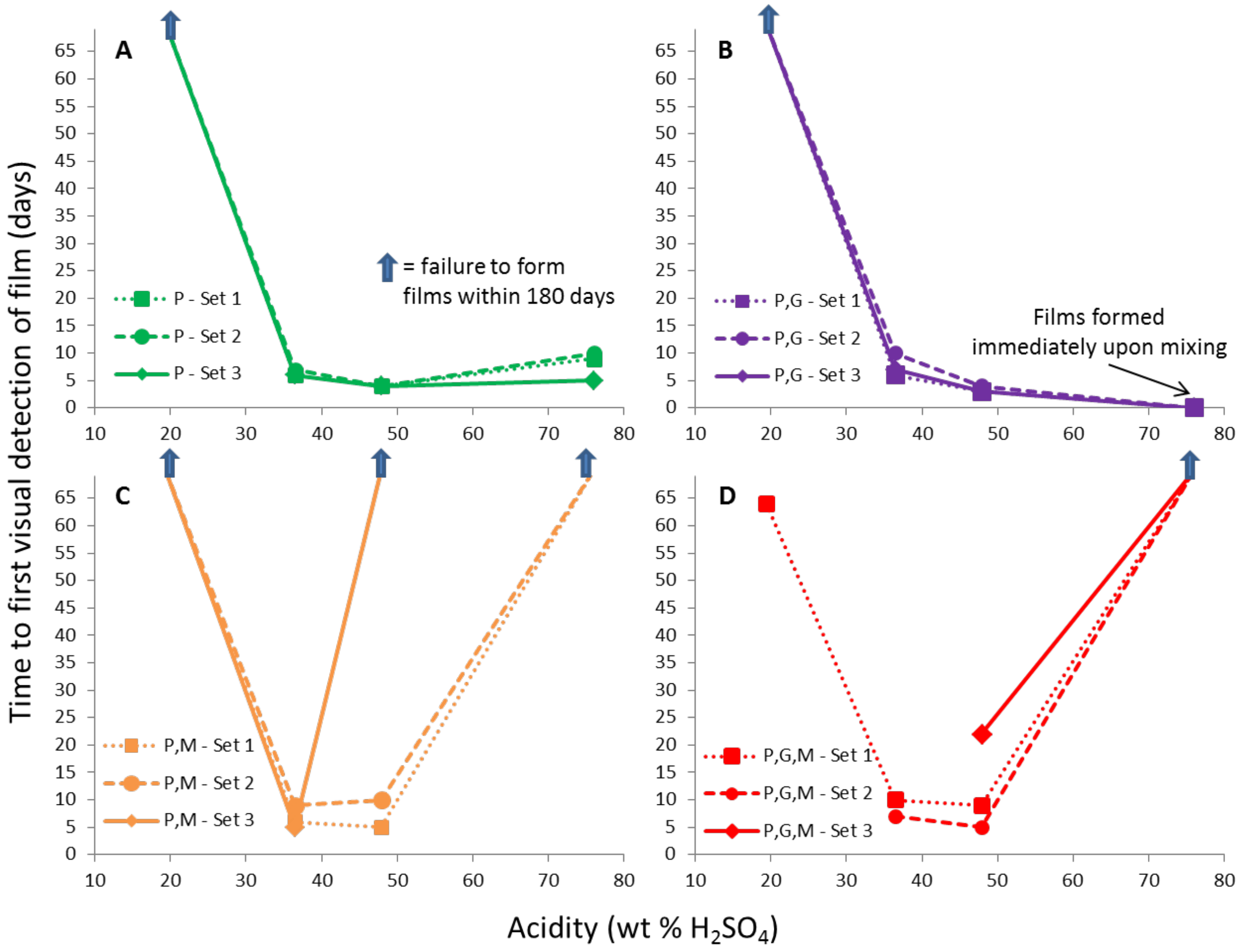

Figure S5. Rates of film formation as a function of acidity (19, 37, 48 and 76 wt % H_2SO_4) for solutions of the following organic mixtures (0.030 M in each organic) stored at room temperature under constant fluorescent room light. A: propanal (P); B: propanal and glyoxal (P,G); C: propanal and methylglyoxal (P,M); D: propanal, glyoxal and methylglyoxal (P,G,M). Rates are expressed as the number of days between mixing and the first detection of a surface film during daily observations. Experiments were performed in three sets. For each set all solutions were made on the same day and stored together and therefore experienced the same lighting conditions, fluctuations in room temperature and handling during observation. Data from the same set are connected by lines to guide the eye in detecting trends. Samples that did not form films within the 180 day duration of the experiments are indicated with blue arrows pointing off scale. Variability between sets is most likely due to differences in the gentle movement of the samples that was required to detect films during daily visual observations. Missing data points (P,M – Set 3 and P,G,M – Sets 2 and 3) indicate samples that were discarded when they were inadvertently tipped over during observation before a film had formed. Note that films formed in the dark showed no significant trend when compared to these films formed under fluorescent room light since formation rates rarely differed by more than a few days.

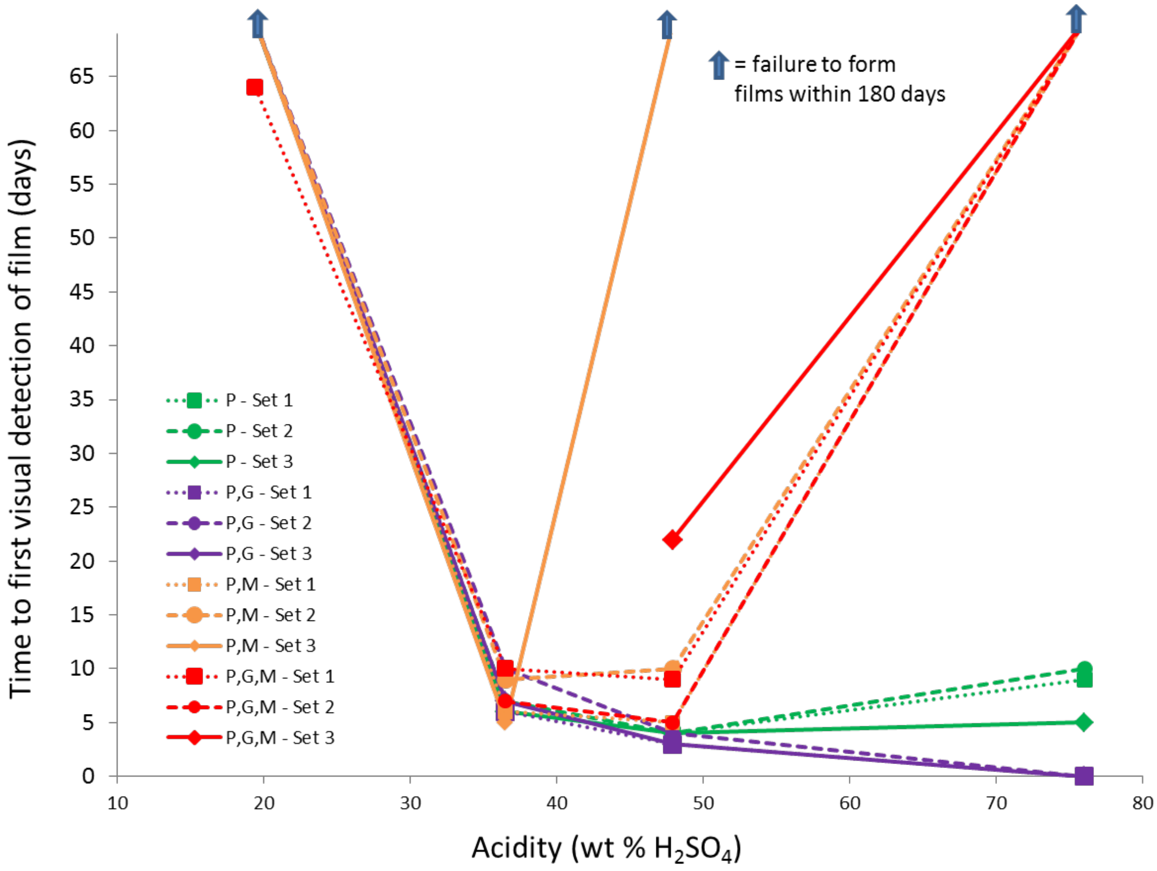

Figure S6. Rates of film formation as a function of acidity from Figure S5 combined into one graph in order to facilitate comparisons among organic mixtures. See caption to Figure S5 for details. Note, in particular, that films form more slowly when methylglyoxal is added by noting that films form more slowly on P,M than on P samples and more slowly on P,G,M than on P,G samples despite the increased organic content in each case.
